# Standardized Non-surgical Rhinoplasty with Hyaluronic Acid: Consistent Outcomes Across Injectors with Varying Experience

**DOI:** 10.1007/s00266-026-05723-4

**Published:** 2026-03-11

**Authors:** Marcelo Germani, Pietra Roschel, Victor Rogerio, Victor R. M. Munoz-Lora

**Affiliations:** 1 Department of Biological Sciences, Bauru School of Dentistry, University of São Paulo, Bauru, Brazil; 2 Department of Biochemistry, Paulista School of Medicine, Federal University of São Paulo, São Paulo, Brazil; 3 Private Office, São Paulo, Brasil; 4 Department of Facial Aesthetics, Guarulhos University, São Paulo, Brazil; 5 HOF Pro Academy, Rio Verde, Goiás Brazil

**Keywords:** Rhinoplasty, Fillers, Hyaluronic acid, Safety, Experience

## Abstract

**Background:**

Non-surgical rhinoplasty using hyaluronic acid (HA) fillers has become an increasingly popular alternative to surgical approaches. However, concerns persist regarding adverse events, particularly when procedures are performed by less experienced injectors.

**Objectives:**

This study aimed to evaluate perceived outcomes of non-surgical rhinoplasty with HA according to the injector’s clinical experience level, and to investigate whether experience influences aesthetic satisfaction, safety, product volume used, or perceived treatment duration.

**Methods:**

This retrospective, single-center study analyzed 59 patients who underwent non-surgical rhinoplasty using HA, performed by 53 injectors with varying clinical experience levels in non-surgical rhinoplasty. Data were collected on age, BMI, volume of HA injected per nasal subunit, injector experience, aesthetic satisfaction using the Global Aesthetic Improvement Scale (GAIS), and perceived duration of results. Statistical analyses included Kruskal–Wallis tests, Spearman correlations, linear regression, and ordinal logistic regression.

**Results:**

The mean total volume of HA injected per procedure was 0.45 (± 0.15) mL, with no significant differences across experience levels (*p* = 0.374). The overall median GAIS score was 1.0 (IQR: 1.0), indicating improvement, with significantly higher satisfaction among patients treated by more experienced injectors (*p* < 0.001). Perceived duration of results was 6–9 months with no significant differences by injector experience (*p* = 0.117). No serious or vascular adverse events were reported.

**Conclusions:**

Non-surgical rhinoplasty with HA may provide favorable outcomes across injector experience levels when performed with a standardized technique and an appropriate product. Greater clinical experience correlated with higher patient satisfaction but not with filler volume or duration of effect.

**Level of Evidence IV:**

This journal requires that authors assign a level of evidence to each article. For a full description of these Evidence-Based Medicine ratings, please refer to the Table of Contents or the online Instructions to Authors www.springer.com/00266.

## Introduction

Hyaluronic acid (HA) injections remain one of the most frequently performed non-surgical aesthetic procedures worldwide, ranking second in global volume according to the 2024 International Survey on Aesthetic/Cosmetic Procedures by the International Society of Aesthetic Plastic Surgery [1]. Among the various indications for HA, non-surgical rhinoplasty—often referred to as nasal bioplasty—has emerged as a popular yet technically challenging application. The procedure offers a minimally invasive alternative to surgical rhinoplasty, providing immediate results, low downtime, and reversibility, making it an attractive option for both patients and practitioners [2].

Despite the various advantages associated with the procedure, healthcare professionals—particularly those with less experience—often express concern about the potential for irreversible adverse events, especially when treating the nasal region [3]. In most cases, such events are associated with professional negligence often resulting from improper technique—rather than the injector’s lack of clinical experience [4, 5].

Recent observational studies have reinforced the safety profile of non-surgical rhinoplasty, with large-scale analyses reporting only minimal adverse events, such as transient edema and bruising, as well as complete resolution of more serious complications [6, 7]. However, these investigations emphasize that practitioner experience and injection technique are critical factors for the success of the procedure.

Nonetheless, few studies have objectively examined whether the use of a standardized technique, together with an appropriate filler, can reduce variability in outcomes across injectors with different levels of clinical experience. Furthermore, recent prospective research using validated instruments such as FACE-Q has demonstrated that patient-reported outcomes, including satisfaction and perceived quality of life, are essential components in evaluating the long-term impact of non-surgical rhinoplasty [8].

In this context, the aim of this study was to retrospectively evaluate whether a standardized non-surgical rhinoplasty technique using HA could deliver consistent, patient-perceived outcomes across injectors with varying levels of experience, thereby testing the replicability of a protocol-based approach in clinical practice.

## Material and Methods

### Study Design

This retrospective, single-center study was conducted between April 2022 and December 2024. The study was approved by the local Research Ethics Committee of Guarulhos University under protocol number CAAE – 83966024.5.0000.5506. All procedures were performed in accordance with Good Clinical Practice guidelines, and written informed consent was obtained from all patients prior to inclusion.

### Study Sample

Fifty-nine Brazilian patients (n = 59) of multi-ethnic background, aged between 20 and 60 years, without a history of prior non-surgical rhinoplasty with HA or nasal trauma were included in the study. Eligibility criteria required complete documentation of the procedure and follow-up, as well as signed informed consent authorizing the use of anonymized clinical data for research purposes. Records with incomplete information or any restriction on data use were excluded.

Fifty-three licensed aesthetic practitioners, participating in structured clinical training activities at our institution, performed the procedures and completed structured forms reporting their clinical experience in nasal bioplasty as well as technical details related to the interventions. This setting allowed us to evaluate the replicability of a standardized technique across injectors with heterogeneous levels of clinical experience. All procedures were conducted under direct faculty supervision and following the same standardized protocol. Signed informed consents were also provided by the practitioners.

### Non-surgical Nasal Reshaping

Before each procedure, all participants were instructed to wash their faces with running water and a neutral soap, followed by rigorous antisepsis using 2% chlorhexidine digluconate in alcoholic solution (Riohex^®^, Rioquímica, São Paulo, Brazil). Local anesthesia was administered through infraorbital nerve block with 2% lidocaine without vasoconstrictor (DFL^®^, Brazil), which did not directly affect the nasal region in order to avoid procedure-related edema.

**Fig. 1:**
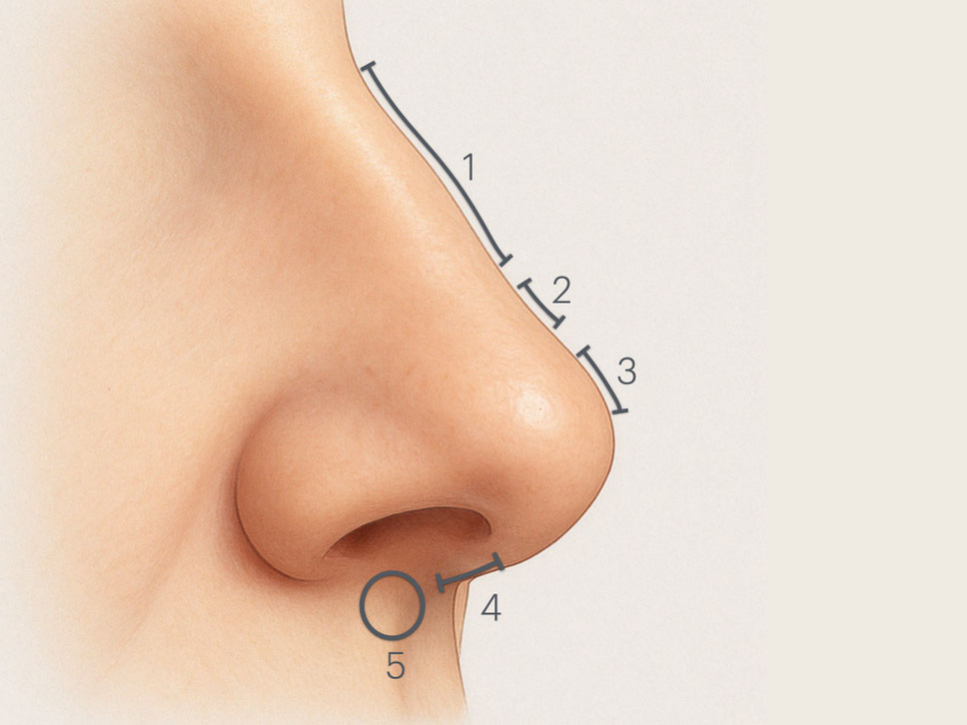
Anatomical segmentation of the nasal subunits evaluated in this study. **1** Dorsum, **2** Supratip, **3** Tip, **4** Columella, and **5** Nasal spine

Treatment area was pre-delineated based on individualized aesthetic assessments, considering facial proportions and specific patient concerns. The volume of filler was carefully adjusted according to the degree of correction required to achieve the desired clinical outcomes. Injections were distributed across distinct anatomical subunits, including: the nasal dorsum (superior longitudinal region of the nasal bridge), the supratip (transition area between the dorsum and nasal tip), the nasal tip (the most anterior and projecting portion of the nose), the columella (the soft tissue bridge between the nostrils), and the nasal spine (the base of the nose, located above the upper lip and between the nostrils) [9] (Fig. 1).

The procedures were performed following the same standardized protocol. For this, all injectors exclusively used 22-gauge blunt-tip cannulas, and the primary technique used was based on the Tip-Up protocol, originally described by Almeida and cols [10], with the adaptation of also including the nasal dorsum in selected cases. The HA used in all treatments was Restylane Lyft^®^ (Galderma, Uppsala, Sweden), a non-animal stabilized hyaluronic acid (NASHA) technology gel characterized by a high elastic modulus (G′ = 545 Pa) and low cross-linking rate (1%) [11]. Although the technique followed predefined anatomical subunits for classification purposes, injectors were free to determine the total volume of HA injected based on individual anatomical assessment and aesthetic goals.

After the procedure, patients received standardized post-treatment instructions, including avoiding manipulation of the treated area, sun exposure, and intense physical activity for the first 24–48 hours. No additional pharmacological interventions were required post-operatively and no reapplications were performed. All patients had direct communication channels with the clinical team via phone or messaging applications to report any adverse effects or complications during the follow-up period.

### Assessments

Data collection was conducted using a structured electronic questionnaire distributed via Google Forms. Patients were asked to report their age and respond to items related to perceived treatment efficacy and satisfaction using the validated Global Aesthetic Improvement Scale (GAIS) [12], which was applied immediately after the procedure. The GAIS consists of a 5-point scale ranging from −2 to +2, where −2 = “Very much worse,” −1 = “Somewhat worse,” 0 = “No change,” 1 = “Somewhat better,” and 2 = “Very much better.”

Additionally, patients were asked to self-report the perceived duration of treatment and possible adverse events, which were continuously monitored through follow-up messages sent via text messaging apps over a one-year period. The perceived duration was categorized into four-time intervals and coded numerically as follows: 1 = up to 3 months, 2 = 3 to 6 months, 3 = 6 to 9 months, and 4 = more than 9 months. Quantitative data regarding the total amount of HA injected into each nasal subunit were compiled from clinical records.

In parallel, injectors completed a separate questionnaire reporting their level of clinical experience with non-surgical rhinoplasty. Experience level in non-surgical rhinoplasty was stratified into four levels and coded numerically: 1 = less than 1 year, 2 = 1 to 3 years, 3 = 3 to 5 years, and 4 = more than 5 years of practice. The number of cases previously performed and the specific time of adoption of the tip-up technique were not available.

All collected data were compiled in spreadsheets and submitted for statistical analysis.

### Statistical Analysis

Statistical analyses were performed using the Jamovi software (The Jamovi Project, version 2.3.28, Sydney, Australia), with the significance level set at *p* ≤ 0.05.

Frequencies were calculated to describe the distribution of injector experience levels, total volume of HA injected, and the perceived duration of treatment. Comparisons between injector experience groups were conducted using the non-parametric Kruskal–Wallis test for three key outcomes: total HA volume injected, GAIS scores, and perceived duration of the treatment. Post hoc comparisons were performed using the Dwass-Steel-Critchlow-Fligner (DSCF) pairwise method.

To assess whether GAIS scores significantly differed from the neutral value (GAIS = 0), a one-sample Wilcoxon signed-rank test was performed. Additionally, Spearman’s rank correlation coefficient was used to explore associations between clinical variables, including age, BMI, total HA volume, injector experience, GAIS scores, and duration.

Finally, a multiple linear regression was used to assess the influence of age, BMI, and injector experience on the total volume of HA injected. Two ordinal logistic regression models were conducted to identify variables associated with GAIS scores and with the perceived duration of treatment, including age, BMI, total HA volume, injector experience, and GAIS.

## Results

### Demographics and Descriptives

This retrospective study included fifty-nine (n = 59) patients of Brazilian multi-ethnic background, with a mean age of 36.0 (± 10.3) years and a mean BMI of 24.6 (± 3.97) kg/m^2^. Treatments were performed by fifty-three different injectors, whose median level of experience was 2.0 (IQR: 2.5), corresponding to 1–3 years of clinical practice. The largest group of injectors was classified as level 1 (< 1 year; 22 injectors, 41.5%), followed by level 4 (>5 years; 14 injectors, 26.4%), level 2 (1–3 years; 14 injectors, 26.4%), and level 3 (3–5 years; 3 injectors, 5.7%).

**Fig. 2:**
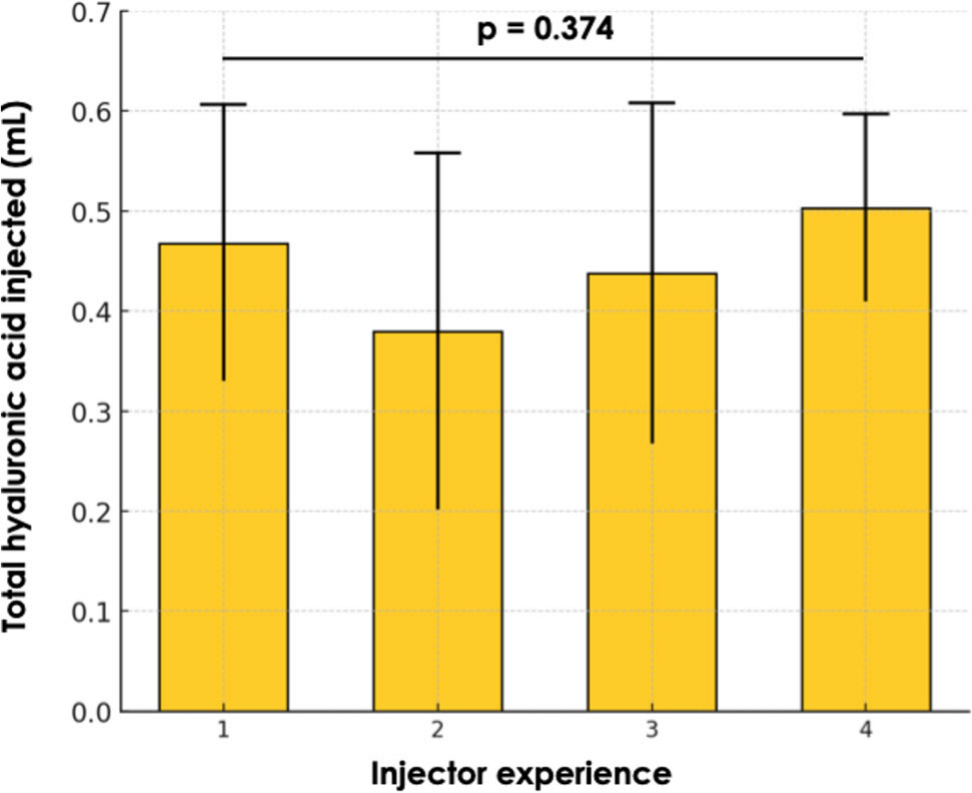
Bar graphs illustrating the mean and standard deviation (SD) of the total volume of hyaluronic acid (HA) injected stratified by injector experience levels (1–4). No statistically significant differences were observed across groups, as determined by the Kruskal–Wallis test

A total mean volume of 0.45 (±0.15) mL of HA was administered per nose. No significant differences were found among injector experience levels in terms of total volume administered (*p* = 0.374). Level 1 injectors administered a mean volume of 0.47 (± 0.14) mL, level 2 administered 0.38 (± 0.18) mL, level 3 administered 0.44 (± 0.17) mL, and level 4 administered 0.50 (± 0.10) mL (Fig. 2).

**Fig. 3:**
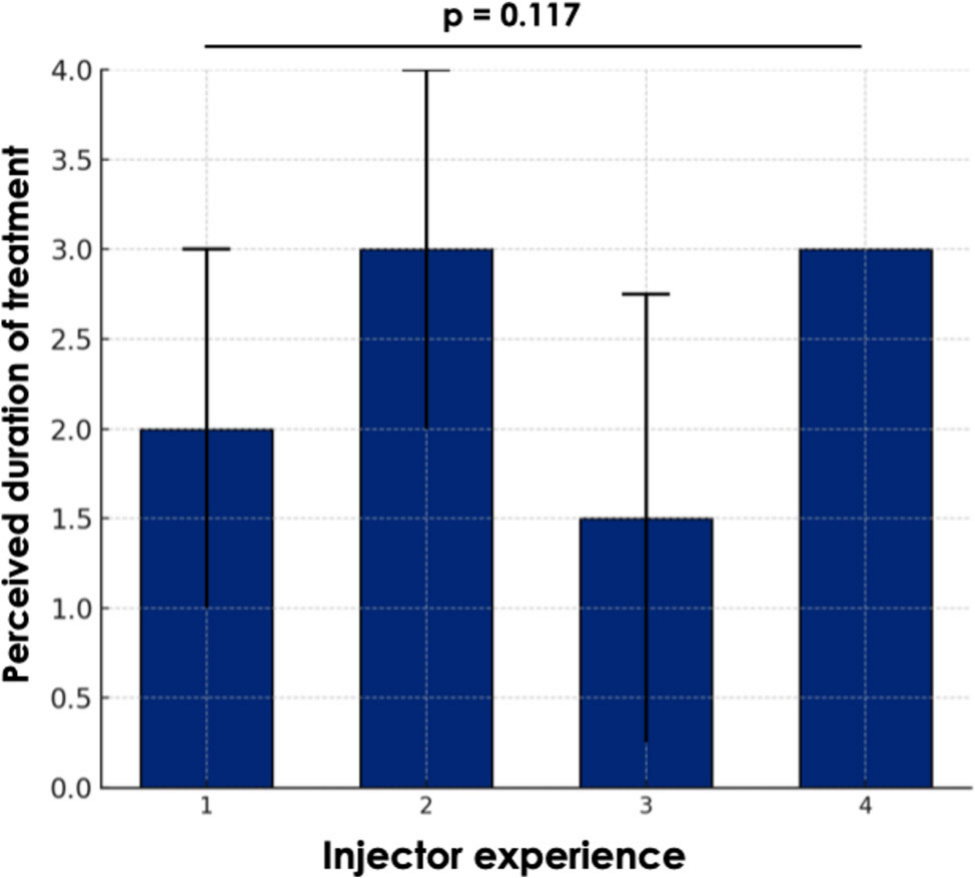
Bar graphs illustrating the median and interquartile range (IQR) of the perceived duration of the treatment stratified by injector experience levels (1–4). No statistically significant differences were observed across groups, as determined by the Kruskal-Wallis test

The overall median perceived duration of treatment effects was 3.0 (IQR: 1.0), suggesting that most patients reported visible results lasting between 6 and 9 months. When stratified by injector experience level, the median durations were: level 1–2.0 (IQR: 1.0), level 2–3.0 (IQR: 1.0), level 3–1.5 (IQR: 1.25), and level 4–3.0 (IQR: 0.0). Although some variation was noted among the groups, these differences were not statistically significant (*p* = 0.117) (Fig. 3).

A complete summary of demographic and clinical characteristics is presented in Table 1.

**Table 1:** Demographic and clinical characteristics of the study sample

Demographics	Value
Patients (n)	59
Age	36.0 (±10.3)
BMI (kg/m^2^)	24.6 (±3.97)
Injectors (n)	53
Experience level	2 (IQR = 2.5)
1	25 (42.4%)
2	15 (25.4%)
3	4 (6.8%)
4	15 (25.4%)
Total volume (mL)	0.45 (±0.15)
1	0.47 (±0.14)
2	0.38 (±0.18)
3	0.44 (±0.17)
4	0.50 (±0.10)
Perceived duration	3 (IQR = 1.0)
1	2 (IQR = 1.0)
2	3 (IQR = 1.0)
3	1.5 (IQR = 1.25)
4	3 (IQR = 0.0)

Age, BMI, and total HA volume are presented as mean ± standard deviation (SD); injector experience levels are shown as absolute and relative frequencies. Total injector experience and perceived duration of treatment are summarized as median and interquartile range (IQR)

### Volume Distribution Within Nasal Subunits

When analyzing the distribution of volumes across different nasal regions (Table 2), the mean volumes were as follows: tip - 0.13 (±0.07) mL, dorsum - 0.14 (±0.08) mL, supratip - 0.02 (±0.04) mL, columella - 0.08 (±0.04) mL, and nasal spine - 0.08 (±0.04) mL. Significant differences were observed between level 2 and level 4 injectors for the columella (*p* = 0.036), nasal spine (*p* = 0.036), and nasal tip (*p* = 0.045).

**Table 2:** Distribution of hyaluronic acid volume injected across nasal subunits according to injector experience level

		Nasal subunits injected				
		Dorsum (mL) Mean ± SD	Supratip (mL) Mean ± SD	Tip (mL) Mean ± SD	Columella (mL) Mean ± SD	Nasal Spine (mL) Mean ± SD
Total volume injected		0.14 (±0.08)	0.02 (±0.04)	0.13 (±0.07)	0.08 (±0.04)	0.08 (±0.04)
Level of experience	1	0.15 (±0.07)	0.01 (±0.03)	0.14 (±0.05)	0.08 (±0.04)	0.09 (±0.03)
	2	0.13 (±0.08)	0.03 (±0.05)	0.10 (±0.08)*	0.06 (±0.05)*	0.06 (±0.05)*
	3	0.18 (±0.03)	0.00 (±0.00)	0.11 (±0.09)	0.08 (±0.05)	0.08 (±0.05)
	4	0.12 (±0.08)	0.01 (±0.04)	0.17 (±0.03)	0.00 (±0.00)	0.00 (±0.00)

Values are presented as mean ± standard deviation (SD), in milliliters. Asterisks (*) indicate statistically significant differences compared to experience level 4 (*p* < 0.05)

### Patient Satisfaction with GAIS

**Fig. 4:**
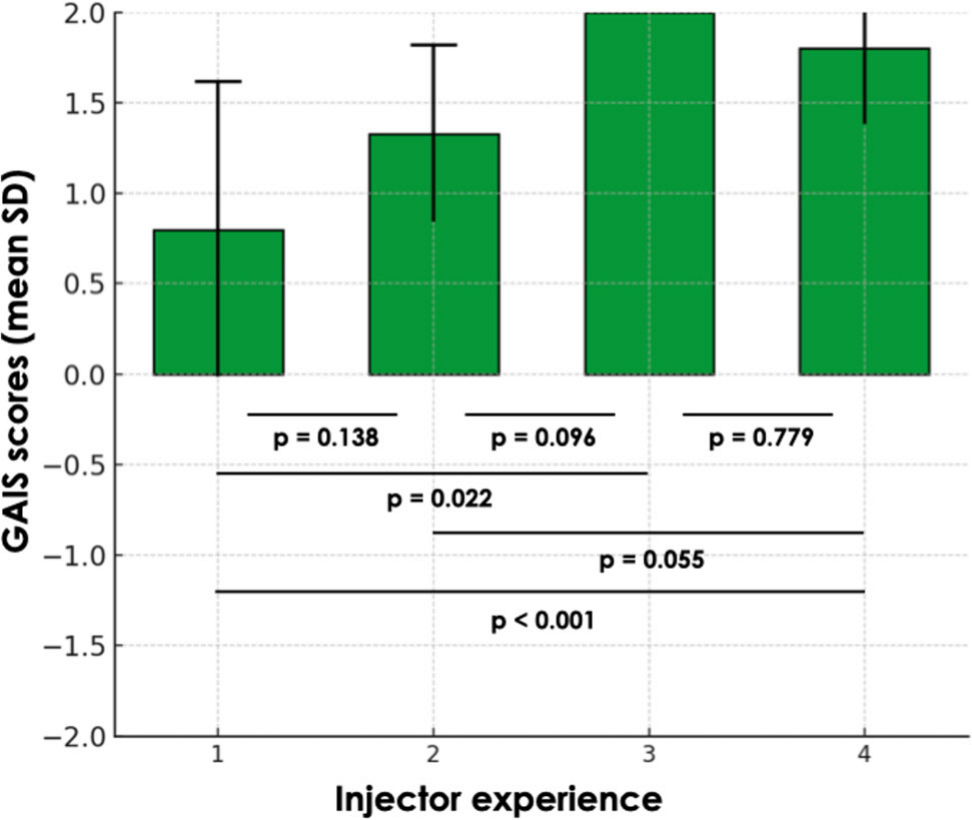
Bar graphs illustrating GAIS scores stratified by injector experience levels (1–4). *p* values lower than 0.05 denote significant differences according to the Kruskal-Wallis test. Mean and standard deviation (SD) were used to increase the readability and understandability of the results

Overall, patients reported favorable aesthetic improvement across all injector experience levels, with a general median score of 1.0 (IQR: 1.0; *p* < 0.001). The median GAIS score for level 1 and level 2 injectors was 1.0 (IQR: 1.0), indicating a perception of “improved” outcomes. For level 3 and level 4 injectors, the median GAIS was 2.0 (IQR: 0.0), corresponding to “much improved,” with no variability observed. A statistically significant difference in GAIS scores was found across the experience levels (*p* < 0.001), with level 1 injectors achieving significantly lower scores compared to level 4 (*p* < 0.001) and level 3 injectors (*p* = 0.022; Fig. 4).

### Exploratory Analysis of Predictive Variables

A multiple linear regression analysis was conducted to evaluate the influence of age, BMI, and injector experience on the total volume of HA injected. The model showed no significant predictive power for any of the independent variables. Age (β = −0.0018; *p* = 0.347), BMI (β = −0.000514; *p* = 0.917), and injector experience (β= 0.0111; *p* = 0.485) showed no significant associations with the total volume of HA injected.

Additionally, the same model was performed to evaluate the influence of GAIS, injector experience, age, BMI, and total volume of HA injected on the perceived duration of treatment, with also no significance among the predictors (all *p* > 0.05).

Finally, an ordinal logistic regression analysis evaluated the effect of age, BMI, total volume of HA injected, and injector experience level on GAIS scores. No significant associations were found for age (*p* = 0.916), BMI (*p* = 0.903), or total volume injected (*p* = 0.266). However, injector experience demonstrated a significant positive effect on GAIS scores (*p* < 0.001), with more experienced injectors showing greater odds of achieving higher satisfaction levels.

Bivariate Spearman correlations were consistent with regression analyses, showing no significant associations between perceived duration and patient age, injector experience, GAIS, or total volume injected. The only strong and significant correlation observed was between injector experience and GAIS scores (rho = 0.600; *p* < 0.001).

Table 3 provides a summary of HA volume, GAIS scores, and perceived duration of treatment sorted by injector experience level, indicating tendencies or similarities across groups.

**Table 3:** Summary of hyaluronic acid (HA) volume used, GAIS scores, and perceived duration of treatment effect according to injector experience level

Experience Level	Volume used (mL) Mean ± SD		GAIS Median ± IQR		Perceived duration Median ± IQR	
1	0.47 (±0.14)		1.0 (1.0)		2.0 (1.0)	
2	0.38 (±0.18)		1.0 (1.0)		3.0 (1.0)	
3	0.44 (±0.17)		2.0 (0.0)*		1.5 (1.25)	
4	0.50 (±0.10)		2.0 (0.0)*		3.0 (0.0)	

Values are presented as mean ± SD for HA volume and as median (IQR) for GAIS and the perceived duration. The direction of the blue arrows indicates tendencies (vertical arrows) or similarities (horizontal arrows) between groups. Asterisks (*) denote statistically significant differences (*p* < 0.05) in GAIS scores compared to level 1

### Adverse Events

**Fig. 5:**
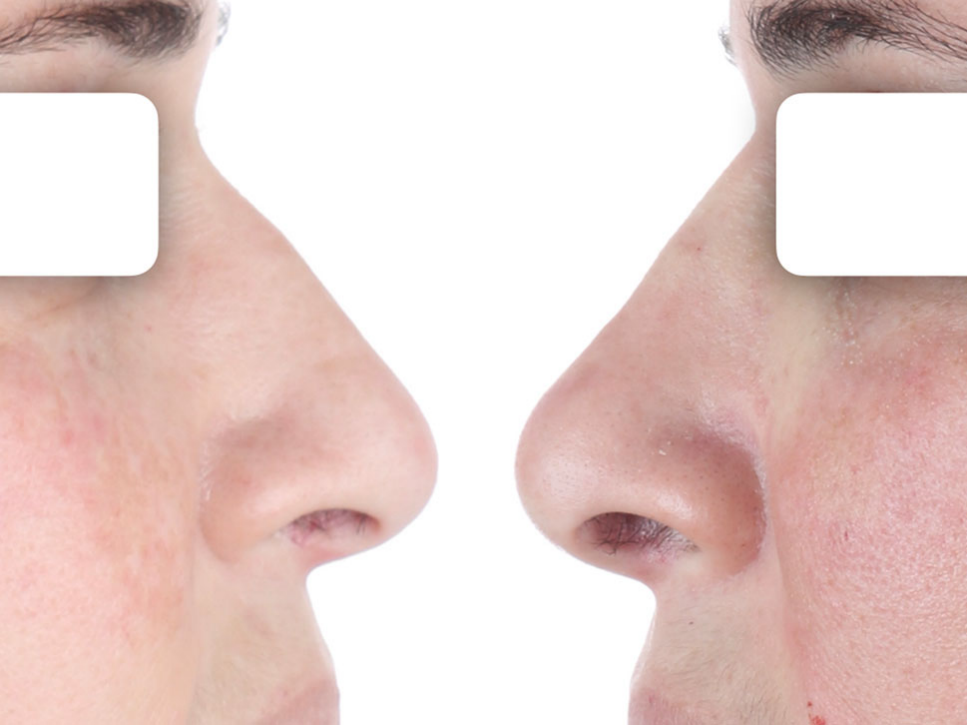
Before-and-after of a 25-year-old female patient (BMI: 25.7) treated with a total of 0.50 mL of hyaluronic acid by a level 1 injector (<1 year of experience). The procedure resulted in a GAIS score of 1, indicating a perception of improvement. The patient reported a perceived duration of effect of 6 - 9 months (score 3)

**Fig. 6:**
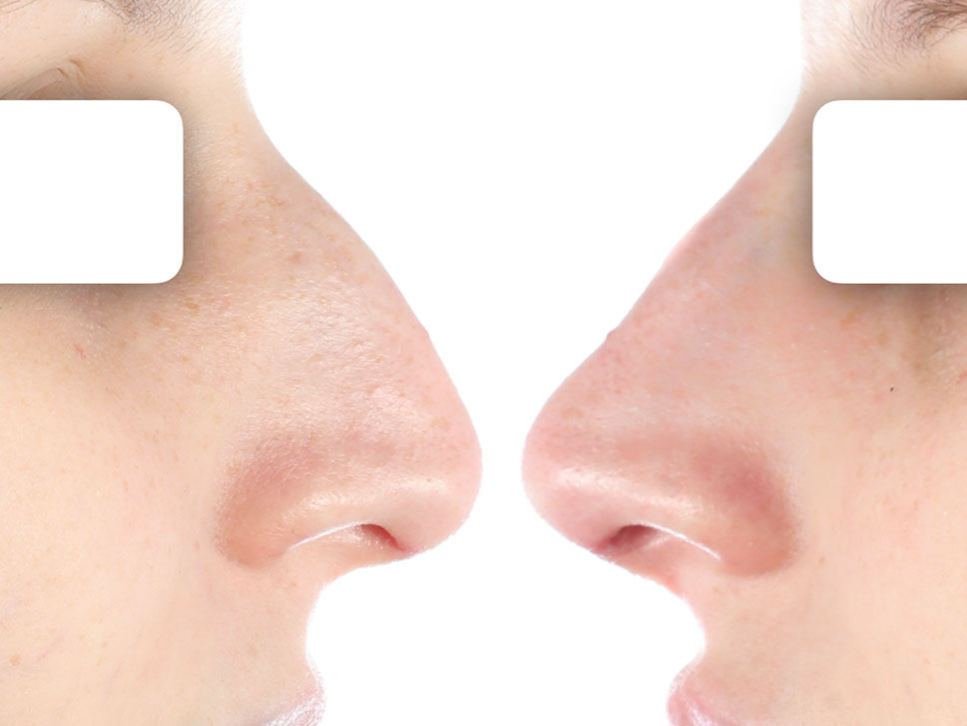
Before-and-after of a 26-year-old female patient (BMI: 19.1) treated with a total of 0.35 mL of hyaluronic acid by a level 2 injector (1–3 years of experience). The procedure resulted in a GAIS score of 1, indicating a perception of improvement. The patient reported a perceived duration of effect greater than 9 months (score 4)

**Fig. 7:**
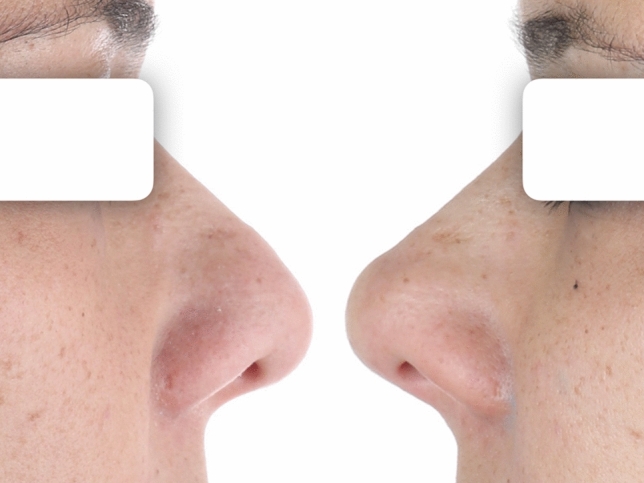
Before-and-after of a 34-year-old female patient (BMI: 25.5) treated with a total of 0.55 mL of hyaluronic acid by a level 3 injector (3–5 years of experience). The procedure resulted in a GAIS score of 2, indicating a perception of very much improved. The patient reported a perceived duration of effect of 6–9 months (score 3)

**Fig. 8:**
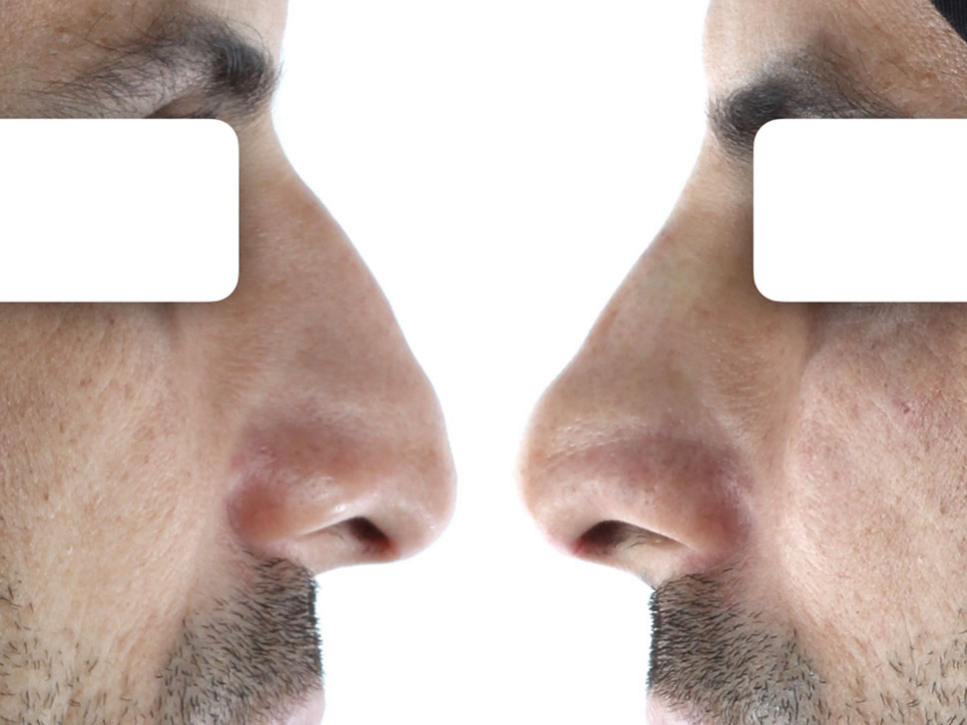
Before-and-after of a 35-year-old male patient (BMI: 24.2) treated with a total of 0.55 mL of hyaluronic acid by a level 4 injector (>5 years of experience). The procedure resulted in a GAIS score of 2, indicating a perception of very much improved. The patient reported a perceived duration of effect of 6 - 9 months (score 3)

During the one-year follow-up period, no serious or vascular adverse events were reported. Only immediate and transient reactions—such as localized edema, mild pain, and ecchymosis—were observed in some cases. All these events resolved spontaneously without the need for any medical intervention (Figs. 5, 6, 7, 8).

## Discussion

The findings of this retrospective study reinforce the clinical applicability of non-surgical rhinoplasty with HA as a minimally invasive, effective, and safe alternative for nasal reshaping. Overall, patient-reported outcomes were favorable across all injector experience levels, as reflected by the GAIS scores. However, a statistically significant correlation was observed between greater clinical experience and higher satisfaction scores, highlighting the importance of technical expertise in the patient’s final aesthetic perception. On the other hand, the absence of statistical variation in the total volume of HA used between groups suggests that the superior outcomes achieved by more experienced professionals may not be attributed to higher volumes of HA, but probably to a more precise assessment, planning, and technical execution of the procedure. Interestingly, all these variables did not affect the perceived duration of the results, independent of the level of experience of the injector. It is important to note that GAIS was applied immediately post-procedure and therefore primarily reflects short-term perception rather than long-term durability.

These findings are in line with recent prospective research using validated patient-reported outcome measures, such as the FACE-Q, which demonstrated that non-surgical rhinoplasty has a positive and lasting impact on patient satisfaction and quality of life [8]. Also, the present results are aligned with the findings of a systematic review that analyzed 16 studies involving non-surgical rhinoplasty with HA dermal fillers [13]. The review highlights the efficacy and safety of the procedure, reporting satisfaction rates exceeding 90% and low incidence of relevant complications. Furthermore, it emphasizes that both, the appropriate selection of HA filler type and the injection technique, are key determinants of the predictability and quality of outcomes as well as the injector experience continues to play a crucial role in mitigating risks such as tissue necrosis and vascular compromise [14]. These points are consistent with the present study, where greater aesthetic satisfaction (as measured by GAIS) was achieved by professionals greater experience in nasal bioplasty, and not necessary because of higher volumes of filler used. Conversely, the perceived duration of treatment effects was not associated with injector experience, suggesting that this outcome may be influenced by a combination of subjective and biological factors, including individual patient expectations and personal aesthetic perception, elements that extend beyond technical competence [15].

The HA filler selected for this study was chosen due to its rheological profile, combining a high elastic modulus (G′) with low cross-linking and reversibility, characteristics considered appropriate for nasal reshaping. [16–18]. This ensured consistency of material properties across all procedures, allowing a more controlled evaluation of the standardized technique, while limiting the interpretation of the findings to the specific HA product used in this study. A previous study evaluated the efficacy and safety of HA with NASHA technology in non-surgical rhinoplasty among 33 women, followed over an eight-month period [19]. The authors reported that 100% of patients showed noticeable aesthetic improvement, with high satisfaction rates sustained throughout follow-up (78.57% at month 8), and no incidence of serious adverse effects. Even though these facts corroborate our findings, the same study reported a mean total volume of 0.84 mL of HA per patient, whereas in our study this value was significantly lower (0.45 mL), representing nearly a 50% reduction in the volume used.

One possible explanation for this discrepancy may lie in the technique used. In the present study, a modified version of the Tip-up technique [10] was applied, focusing on the injection of HA into specific nasal anatomical subunits such as the nasal tip, columella, nasal spine, supratip, and dorsum [20]. In this approach, the nasal tip was treated first, which seems to reduce the need for larger volumes in the columella and nasal spine. This is clinically relevant, as lower volumes may contribute to a more favorable safety profile, as suggested in previous literature [21]. Additionally, the nasal dorsum can be addressed independently from the anterior nasal regions when anatomically indicated. It is important to note that not all subunits require treatment in every patient; injection planning is always based on individual anatomical assessment and aesthetic needs. Furthermore, considering the complexity and different patterns of distribution of nasal arteries [22], the use of a blunt-tip cannula as a standardized delivery device may have contributed to improve safety and better control over product placement, allowing smaller amounts of HA to achieve the desired outcomes. This contrasts with the approach used in the other study, which employed a combination of cannulas and needles [19].

Although standardized training protocols for new aesthetic injectors are still lacking [3], the present findings suggest that the combination of a product with appropriate rheological properties and a standardized injection protocol can help reduce variability of results across injectors with different levels of experience. Despite the limited clinical experience of a significant portion of the injectors included in our study (42.4% with less than one year of practice), outcomes were generally favorable, supporting the feasibility of achieving satisfactory results even among less experienced practitioners when safe strategies and replicable techniques are employed. Importantly, standardization of technique should not be interpreted as simplification of the procedure, as non-surgical rhinoplasty requires detailed anatomical knowledge, careful patient selection, and advanced technical skill, regardless of injector experience level.

This study presents some limitations that should be considered when interpreting the results. First, the absence of a control group limits direct comparisons between different techniques or products. Additionally, the exclusive use of cannulas may restrict the generalizability of the findings to contexts in which needles are commonly used. Injector experience was assessed only by years of practice in non-surgical rhinoplasty, without accounting for case volume or the specific time of adoption of the tip-up technique, which may influence outcomes. Also, the near one-to-one ratio between patients and injectors limits the ability to assess individual injector performance, as case-specific factors may influence subjective outcomes. The findings of this study are limited to primary nasal aesthetic cases and should not be extrapolated to secondary deformities following surgery or trauma, which present distinct anatomical and clinical challenges. Lastly, the assessment of treatment longevity was based on patients’ subjective perceptions rather than objective measurements of volume retention or nasal projection over time. Nevertheless, this study is one of the few investigations to evaluate non-surgical rhinoplasty performed by a large number of injectors (n = 53) using a single standardized protocol, thereby providing insight into the role of technique replicability in patient satisfaction

## Conclusion

This retrospective study suggests that non-surgical rhinoplasty with hyaluronic acid can provide satisfactory outcomes across different injector experience levels when performed with a standardized technique and an appropriate filler. While more experienced practitioners achieved higher satisfaction scores, no differences were observed in filler volume or in the perceived duration of results across groups. These findings highlight the potential of standardized protocols to reduce variability of results between less and more experienced injectors, while reinforcing the importance of structured training and long-term objective assessments

## References

[1] International Society of Aesthetic Plastic Surgery. ISAPS interntional survey on aesthetic/cosmetic procedures in 2024. 2025.

[2] Baser B, Singh P, Shubha P, Roy PK, Chaubey P. Non-surgical rhinoplasty and use of hyaluronic acid based dermal filler-user experience in few subjects. Indian J Otolaryngol Head Neck Surg. 2021;73(1):52–8. 10.1007/s12070-020-02100-8. 33643885 10.1007/s12070-020-02100-8 PMC7881997

[3] Cotofana S, Mehta T, Davidovic K, et al. Identifying levels of competency in aesthetic medicine: a questionnaire-based study. Aesthet Surg J. 2024;44(10):1105–17. 10.1093/asj/sjae096. 38636497 10.1093/asj/sjae096 PMC11403812

[4] Germani M, Alegria P, Giro G, Munoz-Lora VRM. High-dose pulsed hyaluronidase for managing nasal skin necrosis following hyaluronic acid treatment in nasolabial folds: a case report. J Oral Biol Craniofac Res. 2024;14(3):339–41. 10.1016/j.jobcr.2024.04.006. 38699685 10.1016/j.jobcr.2024.04.006 PMC11063503

[5] Vieira MG, Machado-Filho DA, Alcantara AR, Mendonça A, Kim JH, Gonzalez Cortes AR. Clinical management of nasal skin necrosis caused by hyaluronic acid filler. J Craniofac Surg. 2021;32(2):e120–2. 10.1097/SCS.0000000000006847. 33705046 10.1097/SCS.0000000000006847

[6] Jalali A. Nonsurgical rhinoplasty using the hyaluronic acid filler VYC‐25L: safety and patient satisfaction in a retrospective analysis of 492 patients. J Cosmet Dermatol. 2024;23(2):426–33. 10.1111/jocd.15997. 37740484 10.1111/jocd.15997

[7] Giammarioli G, Liberti A. Non‐surgical rhinoplasty technique: an innovative approach for nasal reshaping with hyaluronic acid fillers. J Cosmet Dermatol. 2023;22(7):2054–62. 10.1111/jocd.15669. 36751855 10.1111/jocd.15669

[8] Lombardo GAG, Melita D, Stivala A, Cuomo R, Tamburino S. Assessing the long-term impact of non-surgical rhinoplasty on patient satisfaction and quality of life: a prospective study using FACE-Q. Aesthet Plast Surg. 2025. 10.1007/s00266-024-04644-4. 10.1007/s00266-024-04644-4 39747421

[9] Bertossi D, Lanaro L, Dorelan S, Johanssen K, Nocini P. Nonsurgical rhinoplasty: nasal grid analysis and nasal injecting protocol. Plast Reconstr Surg. 2019;143(2):428–39. 10.1097/prs.0000000000005224. 30531619 10.1097/PRS.0000000000005224

[10] Almeida C, Rogerio V, Giro G, Munoz-Lora V, Germani M. Tip up - simplified technique for non-surgical rhinoplasty: a case series. J Oral Biol Craniofac Res. 2025;15(2):355–8. 10.1016/j.jobcr.2025.01.013. 40303842 10.1016/j.jobcr.2025.01.013 PMC12039370

[11] Fagien S, Bertucci V, von Grote E, Mashburn JH. Rheologic and physicochemical properties used to differentiate injectable hyaluronic acid filler products: Plast Reconstr Surg. 2019;143(4):707e–20e. 10.1097/PRS.0000000000005429. 30921116 10.1097/PRS.0000000000005429 PMC7597953

[12] Germani M, Munoz-Lora VRM, Carnevali ACN, Geroldo AM, Teixeira FF, Giro G. Is more always better? A randomized comparative clinical trial about the impact of polydioxanone threads quantity for facial lifting. Aesthet Surg J Open Forum. 2025;7:ojaf002. 10.1093/asjof/ojaf002. 40236886 10.1093/asjof/ojaf002 PMC11997779

[13] Al-Taie DS, AlEdani EM, Gurramkonda J, et al. Non-surgical rhinoplasty (NSR): a systematic review of its techniques, outcomes, and patient satisfaction. Cureus. 2023. 10.7759/cureus.50728. 38234960 10.7759/cureus.50728 PMC10792339

[14] Kurkjian TJ, Ahmad J, Rohrich RJ. Soft-tissue fillers in rhinoplasty. Plast Reconstr Surg. 2014;133(2):121e–6e. 10.1097/01.prs.0000437246.61294.33. 24150121 10.1097/01.prs.0000437246.61294.33

[15] Germani M, Borba PR, Carnevali ACN, et al. Unveiling patient expectations: insights from a cross-sectional study on facial aesthetic treatments. Dermatol Surg. 2024. 10.1097/DSS.0000000000004430. 39584694 10.1097/DSS.0000000000004430

[16] Germani M, De Queiroz MVGB, Yuri De França Shimizu M, et al. Comparative in-vitro degradation of hyaluronic acids exposed to different hyaluronidase enzymes. J Oral Biol Craniofac Res. 2025;15(1):178–82. 10.1016/j.jobcr.2025.01.001. 39897433 10.1016/j.jobcr.2025.01.001 PMC11782997

[17] Bertucci V, Lynde CB. Current concepts in the use of small-particle hyaluronic acid: Plast Reconstr Surg. 2015;136:132S-138S. 10.1097/PRS.0000000000001834. 26441093 10.1097/PRS.0000000000001834

[18] Germani Vieira M, Rogerio V, Roschel P, Rabelo V, Teixeira T, Muñoz-Lora VRM. Myomodulation using hyaluronic acid fillers as an efficient and innovative treatment for gummy smile: a case report. J Oral Biol Craniofac Res. 2022;12(3):376–80. 10.1016/j.jobcr.2022.04.009. 35586484 10.1016/j.jobcr.2022.04.009 PMC9108743

[19] Nikolis A, Enright KM, Nguyen Q, Sinno HH, Cotofana S. A prospective clinical trial evaluating the efficacy and safety of non-animal stabilized hyaluronic acid injections for non-surgical rhinoplasty. Plast Surg (Oakv). 2025;33(1):97–106. 10.1177/22925503231184263. 39876851 10.1177/22925503231184263 PMC11773380

[20] Segreto F, Marangi GF, Cerbone V, Alessandri-Bonetti M, Caldaria E, Persichetti P. Nonsurgical rhinoplasty: a graft-based technique. Plast Reconstr Surg Glob Open. 2019;7(6):e2241. 10.1097/GOX.0000000000002241. 31624669 10.1097/GOX.0000000000002241 PMC6635207

[21] Heydenrych I, Kapoor KM, De Boulle K, et al. A 10-point plan for avoiding hyaluronic acid dermal filler-related complications during facial aesthetic procedures and algorithms for management. CCID Curr Clin Infect Dis. 2018;Volume 11:603–11. 10.2147/CCID.S180904. 10.2147/CCID.S180904 PMC6257077 30538521

[22] Cai B, Yuan R, Zhu GZ, et al. Deployment of the ophthalmic and facial angiosomes in the upper nose overlaying the nasal bones. Aesthet Surg J. 2021;41(12):NP1975–85. 10.1093/asj/sjab003. 33421060 10.1093/asj/sjab003

